# Treatment of Epilepsy

**Published:** 1878-06

**Authors:** 


### EXCERPTA.

---

TREATMENT OF EPILEPSY.—Dr. Shultz records, in the *Berliner klinische Wochenschrift*, the case of a young man, eighteen years of age, the subject of epileptic attacks, which always came on at a certain hour in the day. It mattered not what he might at that time be doing, the attack never failed. It was always preceded by an aura which lasted five or six minutes, and was followed by a sleep of several hours' duration. Quininine large and small doses, strychnine, belladonna, nitrate of silver, morphia, chloral, etc., were all administered without result; the attacks continued to recur at the fixed hour, and even occurred during sleep induced by chloral. Coming at this time under Schultz's care, he determined to test Nothnagle's treatment, and administered a teaspoonful of ordinary salt during the aura. This did not at first prevent the attack, but when on the following day a heaping tablespoonful of salt was given at the very beginning of the aura, no attack took place. For one week the dose was administered at the usual time, although no aura was perceived. At the date of Schultz's report (seven weeks afterward) no attacks had been observed, though previous to the treatment, the patient had had them for one hundred and thirty-four days in succession.

